# Integrated Transcriptome–Translatome Analysis Reveals Translational Buffering in the Liver of Chickens Infected with Fowl Adenovirus Serotype 4 Strain GXYL-03, a Strain with Reduced Virulence

**DOI:** 10.3390/microorganisms14102226

**Published:** 2026-10-02

**Authors:** You Wei, Qing Fan, Zhixun Xie, Xiaofeng Li, Sheng Wang, Bingyi Yang, Yanfang Zhang, Lijun Wan, Aiqiong Wu, Hongyu Ren, Zuoxin Chen, Liji Xie, Meng Li, Tingjun Hu, Sisi Luo

**Affiliations:** 1 Guangxi Key Laboratory of Animal Breeding, Disease Control and Prevention, College of Animal Science and Technology, Guangxi University, Nanning 530004, China; weiyou0909@163.com (Y.W.); fanqing1224@126.com (Q.F.); 2 Key Laboratory of China (Guangxi)-ASEAN Cross-Border Animal Disease Prevention and Control, Ministry of Agriculture and Rural Affairs, Guangxi Key Laboratory of Veterinary Biotechnology, Guangxi Veterinary Research Institute, Nanning 530001, China; xiezhixun@126.com (Z.X.); lixiaofeng2003@126.com (X.L.); wangsheng1021@126.com (S.W.); yangby1999@126.com (B.Y.); zhangyanfang409@126.com (Y.Z.); wanlijun0529@163.com (L.W.); wuaiqiong88@163.com (A.W.); renhongyu328@126.com (H.R.); zuoxinchen86@gmail.com (Z.C.); xie3120371@163.com (L.X.); mengli4836@163.com (M.L.)

**Keywords:** fowl adenovirus serotype 4 (FAdV-4), strain with reduced virulence, polysome profiling, translation efficiency, translational buffering

## Abstract

Fowl adenovirus serotype 4 (FAdV-4) causes hydropericardium-hepatitis syndrome (HHS) and has resulted in severe economic losses to the poultry industry in China since 2015. In this study, we isolated a naturally occurring strain with reduced virulence designated GXYL-03, which caused no gross or histological HHS lesions in 4-week-old specific pathogen-free (SPF) chickens infected at 10^6^ TCID_50_, whereas clinical signs and partial mortality were observed at higher doses of 10^7^–10^8^ TCID_50_. After a single immunization with 5 × 10^4^ TCID_50_ of GXYL-03, all ten immunized SPF chickens survived throughout the 14-day observation following lethal GX2017-005 challenge, whereas all ten challenge-control chickens died. Integrated transcriptome and translatome analyses were performed on the livers of chickens infected with FAdV-4 strains of differing virulence by RNA sequencing (RNA-seq) and polysome profiling, with three biological replicates per group. The polysome/non-polysome (P/NP) ratio did not differ significantly among groups (*p* > 0.05), indicating stable global translation flux. The GXYL-03 group had 2,131 transcriptomic differentially expressed genes (DEGs) but only 71 translatomic DEGs; the GX2017-005 group had 7767 and 148, respectively, indicating that only 3.3% and 1.9% of transcriptional perturbations were transmitted to translation, revealing a pronounced translational buffering effect. Translation efficiency (TE) changes were predominantly suppressive, and downregulated genes accounted for 58.6% and 61.8% of TE changes in the two strains. The functional enrichment of genes whose expression was synchronously upregulated at both levels (TE+RNA+) revealed that GXYL-03 was enriched mainly in protein-coding genes involved in cellular stress, DNA damage repair, vesicle transport, and protein homeostasis (*HSPA2*, *HSPB9*, *FANCD2*, *EHD2*, and *Vault*), whereas GX2017-005 was enriched in liver injury-related genes (*CD74*, *ATG2B*, *FKBP5*, *ND1*, and *COX1*) that were not differentially expressed in GXYL-03. This study provides insights into the development of FAdV-4 strains with reduced virulence and virus–host interactions.

## 1. Introduction

Fowl adenovirus serotype 4 (FAdV-4) belongs to the genus *Aviadenovirus* and species *Aviadenovirus* C within the family Adenoviridae [1,2]. It is a nonenveloped virus possessing a linear double-stranded DNA genome of 43–46 kb in length. FAdV-4 has been identified as the causative agent of hydropericardium hepatitis syndrome (HHS) [3]. HHS was first documented in the Ankara region of Pakistan in 1987, followed by outbreaks in Australia, India, Japan, South Korea and other regions [4,5,6]. Since 2015, sporadic cases have emerged in China and have rapidly spread across major poultry-producing provinces including Shandong, Henan, Yunnan, Jilin, Guangdong and Guangxi [7,8,9]. HHS has become one of the major emerging diseases affecting young chickens in recent years. HHS induced by FAdV-4 causes mortality and growth retardation in broilers and laying hens, with clinical mortality rates ranging from 20% to 80%. In addition, FAdV-4 damages the immune organs of surviving chickens, resulting in impaired flock immunity and reduced feed conversion ratios [10]. Collectively, these effects cause substantial economic losses to the poultry industry. Accordingly, the prevention and control of HHS have attracted considerable attention in poultry production and clinical prophylaxis in recent years.

Although all FAdV-4 strains are classified within the same serotype, the pathogenicity of isolates from different geographic regions varies considerably. The international reference strain KR5, an FAdV-4 isolate originating from South Korea, causes only mild clinical signs upon infection and is recognized as a low-virulence strain [11]. The nonpathogenic Canadian isolate ON1 does not induce HHS but elicits neutralizing antibodies; therefore, it is termed as a potential vaccine vector backbone [12,13]. In contrast, the Mexican isolate MX-SHP95 causes 100% mortality in 1-day-old specific pathogen-free (SPF) chickens at an inoculation dose of 2.5 × 10^5^ TCID_50_, representing a typical highly pathogenic strain [14]. The highly pathogenic FAdV-4 strains that emerged and spread widely in China in 2015 belong to a novel genotype, characterized by a hallmark 1966-bp genomic deletion between open reading frame (ORF)42 and ORF43 [15]. Although genomic comparisons have identified multiple genetic locus differences between highly pathogenic and low-virulence strains [16,17,18], how these differences ultimately translate into divergent pathogenic phenotypes through virus–host interactions remains unclear.

Indeed, the virulence of a viral strain depends not only on its own genomic features but also on the manner and extent to which the host gene expression network is perturbed during infection. In recent years, RNA sequencing (RNA-seq) has been widely employed to dissect host responses following FAdV-4 infection, revealing the dysregulation of multiple pathways including innate immune responses, apoptosis, inflammatory reactions, and metabolic disturbances, thereby providing important insights into the pathogenic mechanisms of FAdV-4. Zhang et al. [19] analyzed transcriptomic data from Leghorn male hepatoma (LMH) cells at 12, 24, and 48 h post-FAdV-4 infection and reported that viral infection activated immune-related signaling pathways, including the Toll-like receptor (TLR) pathway, and confirmed that MyD88 mediates FAdV-4-induced inflammatory responses. Ren et al. [20] used RNA-seq to identify differentially expressed genes (DEGs) in the livers of FAdV-4-infected chickens at 7, 14, and 21 days postinfection (dpi), and found that during the late stage of infection, host DEGs were enriched mainly in the peroxisome proliferator-activated receptor (PPAR), Notch, and TLR signaling pathways. Chen et al. [21] reported that in chicken livers at 2 days post-FAdV-4 infection, the virus exploits the phagosome pathway to facilitate infection. However, these studies focused nearly exclusively on the transcriptional level. Importantly, mRNA abundance does not equate to protein expression levels, and changes at the transcriptional level often fail to accurately reflect the functional output of genes. Accumulating evidence indicates that viral infection can broadly regulate host gene expression at the translational level, resulting in transcription–translation discordance as well as translational buffering [22,23], whereby alterations in total mRNA abundance are counter-compensated by changes in translation efficiency (TE), maintaining relatively stable levels of polysome-associated mRNA and, consequently, protein output. Therefore, RNA-seq data alone may severely underestimate or misrepresent the true extent of viral regulation of host gene expression.

Polysome profiling is a classic technique for dissecting translational regulation [24]. By separating actively translating mRNAs associated with polysomes through sucrose density gradient centrifugation and coupling this with high-throughput sequencing, genome-wide quantification of the translational status of genes can be achieved. Compared with RNA-seq, this technique directly quantifies actively translating mRNAs, allows the calculation of TE, and identifies transcription–translation discordance as well as translational buffering [23,25], thereby compensating for the limitation of measuring only total mRNA abundance. However, the application of polysome profiling in FAdV-4 infection studies has not yet been reported, and the characteristics of host translational responses following FAdV-4 infection remain entirely unknown.

In the present study, we isolated and identified an FAdV-4 strain from diseased chickens, designated as GXYL-03. Genomic sequencing and SPF chicken experiments confirmed that this strain exhibits low pathogenicity and that immunization with it confers complete protection against challenge with the highly pathogenic strain GX2017-005. Based on these findings, we combined RNA-seq and polysome profiling to systematically compare host hepatic gene expression responses at both the transcriptional and translational levels following infection with strains GXYL-03 and GX2017-005, with a focus on dissecting the translational buffering effect and identifying differentially enriched functional genes between the two strains. This study aims to provide new insights into the development and application of FAdV-4 strains with reduced virulence and the elucidation of virus–host interaction mechanisms.

## 2. Materials and Methods

### 2.1. Statement of Experimental Animals and Ethics

SPF White Leghorn chicken eggs were purchased from Beijing Boehringer Ingelheim Vital Biotechnology Co., Ltd. (Beijing, China). The eggs were incubated at 37 °C, and the hatched chickens were raised in negative-pressure SPF isolators until they reached 4 weeks of age to evaluate the pathogenicity and immune protective efficacy of the FAdV-4 strain. All animal experiments were approved by the Animal Ethics Committee of Guangxi Veterinary Research Institute (Approval No. GXV-2025-034) and conducted in accordance with the guidelines established by the committee.

### 2.2. Isolation and Identification of Strain GXYL-03

Tissue samples were collected from 5-week-old laying hens showing reduced feed intake and diarrhea at a commercial laying-hen farm in Guangxi, Southwest China, in 2024. No obvious organ lesions were observed upon necropsy. Liver tissues were aseptically collected and thoroughly homogenized in phosphate-buffered saline (PBS). After three repeated freeze–thaw cycles, the homogenate was centrifuged at 12,000× *g* for 10 min. The supernatant was harvested and filtered through a 0.22 μm polyethersulfone (PES) syringe filter for subsequent use.

Total DNA was extracted from the supernatant using an EasyPure^®^ Genomic DNA Kit (TransGen, Beijing, China). Polymerase chain reaction (PCR) was performed on the extracted DNA with FAdV-4 Hexon-specific primers: forward, 5′-CGAGGTCTATACCAACACGAGCA-3′; reverse, 5′-TACAGCAGGTTAATGAAGTTATC-3′ [26]. The thermal profile consisted of initial denaturation at 98 °C for 5 min, 30 cycles of 98 °C for 10 s, 55 °C for 5 s, and 72 °C for 40 s and a final extension at 72 °C for 8 min.

Sterile-filtered liver supernatant was inoculated onto chicken embryo liver (CEL) cells for three serial blind passages. The harvested virus stock was inoculated onto CEL cell monolayers in 6-well plates and incubated at 37 °C for 1 h to allow viral adsorption. Following inoculum removal, the cells were overlaid with Dulbecco’s modified Eagle’s medium (DMEM; Gibco, Waltham, MA, USA) supplemented with 1% low-melting-point agarose (Promega, Madison, WI, USA) and 2.5% fetal bovine serum (FBS; Gibco, USA). After 5–6 days of incubation, single plaques were picked and propagated in fresh CEL cells, with three independent rounds of plaque purification performed overall. The purified viral isolate was designated GXYL-03, propagated to generate a seed lot, and stored at −80 °C. Subsequently, the identity of the isolate was confirmed by indirect immunofluorescence assay (IFA) using an in-house prepared monoclonal antibody against the FAdV-4 fiber-2 protein as the primary antibody, and its TCID_50_ was determined.

### 2.3. Pathogenicity Assay of Strain GXYL-03

Purified GXYL-03 was inoculated into CEL cells and chicken LMH at a multiplicity of infection (MOI) of 0.5. Cells were cultured at 37 °C with 5% CO_2_, and the cytopathic effect (CPE) was observed and recorded daily. 10-day-old SPF chicken embryos were inoculated with 10^5^ TCID_50_ of GXYL-03 via the chorioallantoic membrane (CAM). The embryos were further incubated for 96 h, and embryonic lesions and mortality were monitored and documented.

Four-week-old SPF chickens were randomly allocated into eight groups (10 birds per group). Chickens in the infection groups received intramuscular inoculation with strain GXYL-03 at serial doses of 10^2^ to 10^8^ TCID_50_, whereas those in the control group were injected with an equivalent volume of PBS. All the animals were raised in individual SPF isolators and monitored daily over a 14-day observation period. Clinical manifestations and mortality were recorded to assess the pathogenicity of strain GXYL-03.

An additional ten 4-week-old SPF chickens were intramuscularly inoculated with strain GXYL-03 at 10^6^ TCID_50_, whereas another ten birds injected with an equivalent volume of PBS served as blank controls. All the chickens were euthanized at 5 days postinoculation (dpi). Gross pathological lesions characteristic of FAdV-4 infection in the heart and liver were visually inspected. Tissue samples, including the liver, heart, spleen, kidney, bursa of Fabricius, and thymus were harvested and fixed in 10% neutral buffered formalin for subsequent histopathological analysis.

### 2.4. Immune Protective Efficacy Evaluation of Strain GXYL-03

To assess the protective efficacy of strain GXYL-03 against highly pathogenic FAdV-4, thirty 4-week-old SPF chickens were randomly allocated into three groups of 10 birds each. The birds in the immunization–challenge group were intramuscularly vaccinated with strain GXYL-03 at 5 × 10^4^ TCID_50_ per bird, whereas the challenge control and blank control groups received an equivalent volume of PBS. 14 days postimmunization, the immunization–challenge and challenge control groups were challenged with 5 × 10^3^ TCID_50_ of the virulent FAdV-4 strain GX2017-005. All birds were monitored daily for 14 days postchallenge (dpc), and morbidity and mortality were recorded for each group.

### 2.5. Whole-Genome Sequencing and Analysis of GXYL-03

The full genome of strain GXYL-03 was amplified by segmented PCR using PrimeSTAR^®^ HS High-Fidelity DNA Polymerase (TaKaRa, Kyoto, Japan) with 46 pairs of specific primers spanning the entire FAdV-4 genome [18]. PCR amplicons were separated by 1% agarose gel electrophoresis and gel-purified. Purified fragments were subsequently cloned and inserted into the pMD18-T vector and Sanger sequenced at Beijing Tsingke Biotech Co., Ltd. (Beijing, China), and the resulting sequences were manually assembled into a draft genome. In parallel, the same viral DNA sample was sent to Sangon Biotech (Shanghai) Co., Ltd. (Shanghai, China), for Illumina next-generation sequencing (NGS). The final complete genome was obtained by cross-validation and integration of Sanger and Illumina data.

Full-length nucleotide sequences of strain GXYL-03 and 45 reference FAdV strains covering species A–E retrieved from GenBank were aligned using ClustalW in MEGA 11 [27]. The deduced amino acid sequences of the major structural proteins of GXYL-03, including Penton base, Hexon, Fiber-1, and Fiber-2, were compared with those of FAdV-4 strains of different virulence levels. A neighbor-joining phylogenetic tree was constructed in MEGA 11 [27]. Genetic distances were calculated with the maximum composite likelihood model, and tree reliability was evaluated by 1000 bootstrap replicates.

### 2.6. Translatome and Transcriptome Sequencing of FAdV-4-Infected SPF Chicken Livers

Sixty 4-week-old SPF chickens were randomly divided into three groups (*n* = 20 per group): the GXYL-03 infection group, the GX2017-005 infection group, and the control group. Chicken in the infection groups was intramuscularly injected with 0.5 mL of virus suspension containing 5 × 10^4^ TCID_50_ of FAdV-4, whereas chickens in the control group received an equal volume of PBS. At 3 dpi, all chickens were euthanized and liver tissues were rapidly collected, snap-frozen in liquid nitrogen, and stored at −80 °C until use.

Equal amounts of liver tissue were used to prepare cytoplasmic lysates under nondenaturing conditions. Each lysate was divided into two aliquots: one aliquot was subjected directly to total RNA extraction using TRIzol reagent for transcriptome sequencing, while the other was subjected to polysome profiling. Polysome fractions were isolated by sucrose density gradient centrifugation, and polysome-associated RNA was extracted for translatome sequencing. The quality of the RNA was assessed separately: RNA integrity and potential DNA contamination were evaluated by agarose gel electrophoresis, and purity was determined by measuring the OD_260_/_280_ and OD_260_/_230_ ratios with a NanoPhotometer (Implen GmbH, Munich, Germany). Polysome profiles were generated by measuring the OD_260_ absorbance of each fraction to determine the nucleic acid content. All RNA samples that passed quality control were used for subsequent library construction.

Independent sequencing libraries were constructed from total RNA and polysome-associated RNA, and paired-end sequencing was performed on the Illumina platform. Library construction and sequencing were carried out by LC-Bio Technologies (Hangzhou) Co., Ltd. (Hangzhou, China). Raw reads were subjected to quality control and filtering to obtain high-quality clean reads. Using the chicken reference genome (*Gallus gallus* GRCg7b) as the reference, the clean reads were mapped to the genome with the aligner HISAT2. Raw counts for each gene were summarized, and fragments per kilobase of transcript per million mapped reads (FPKM) values were calculated for gene expression quantification.

### 2.7. Differential Expression and Integrated Transcription–TE Analysis of the Transcriptome and Translatome in FAdV-4-Infected SPF Chicken Livers

Based on the gene raw count matrix, differential expression analysis of the RNA-seq and polysome-seq data was performed separately using the negative binomial model in DESeq2 [28]. Raw counts were normalized by library size, and raw *p* values were adjusted using the Benjamini–Hochberg (BH) method [29] to control the false discovery rate (FDR).

The GXYL-03 and GX2017-005 infection groups were compared with the control group to identify DEGs. The TE was calculated as the ratio of polysome FPKM to total FPKM (P/T) to represent translational activity. A uniform screening threshold was applied to the transcriptomic DEGs, translatomic DEGs, and TE-DEGs: |log2(fold change, FC)| ≥ 1 and adjusted *p* ≤ 0.05, where log2(FC) ≥ 1 was defined as upregulated genes and log2(FC) ≤ −1 as downregulated genes.

For all the detected host genes, an integrated scatter plot was constructed with transcriptomic log2(FC) on the y-axis and the log2 (FC) of the TE on the x-axis. Genes were categorized into four transcriptional–translational coregulation modes (TE+RNA+, TE+RNA−, TE−RNA+ and TE−RNA−) based on the |log2 FC| = 1 threshold, with genes showing no significant changes in either dimension labeled as not significant (NS). Gene Ontology (GO) functional enrichment analysis, focusing on biological process (BP) terms, was conducted for each quadrant to characterize the functional features associated with each regulation mode.

Based on the four-quadrant classification and functional enrichment results, representative genes associated with the immune response, cellular stress, metabolism, and translational regulation were selected for quantitative real-time PCR (qPCR) validation. Total RNA extracted from the same biological samples was reverse-transcribed into cDNA using a reverse transcription kit (TaKaRa, Kyoto, Japan) following the manufacturer’s protocol. qPCR was performed on a QuantStudio™ 5 Real-Time PCR System, Applied Biosystems (Waltham, MA, USA) with SYBR Green chemistry (the primers used are listed in Table 1) using GAPDH and β-actin as dual reference genes. The primers for the target genes were designed in-house based on the corresponding *Gallus gallus* reference sequences in NCBI. The 20 μL reaction mixture contained 2 μL of cDNA, 10 μL of 2× SYBR Green mix, 0.4 μL of each primer (10 μM), and 7.2 μL of ddH_2_O. The cycling conditions were 95 °C for 30 s, followed by 40 cycles of 95 °C for 5 s and 60 °C for 30 s, with a melting curve analysis at the end. Relative gene expression was calculated by the 2^−ΔΔCt^ method [30]. Each group included three biological replicates and three technical replicates. The expression trends from qPCR were compared with those from high-throughput sequencing to confirm data reliability.

## 3. Results

### 3.1. Isolation and Identification of Strain GXYL-03

**Figure 1:**
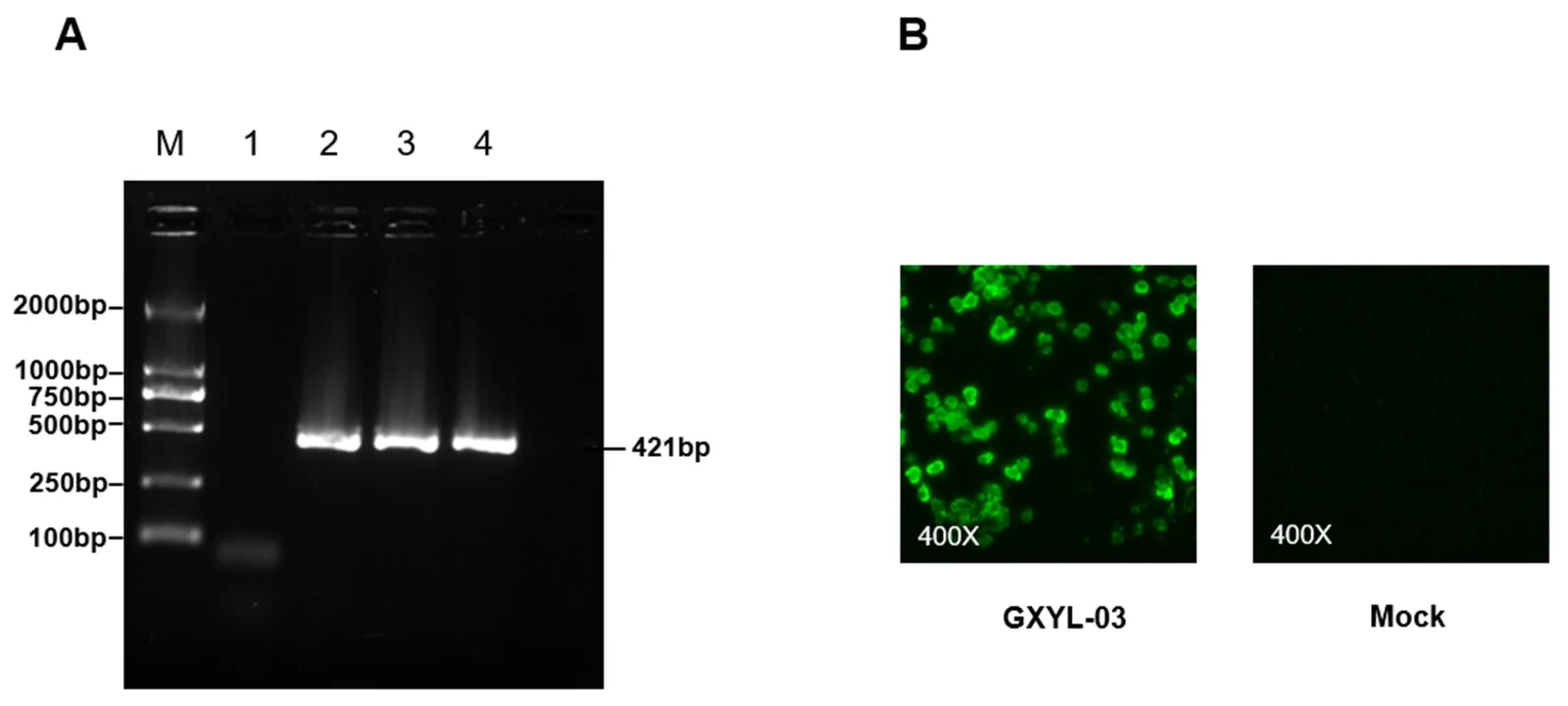
Identification of strain GXYL-03 as FAdV-4. (**A**): PCR identification using FAdV-4-specific primers. M: DL2000 DNA marker; Lane 1: negative control; lane 2: positive control; lanes 3–4: GXYL-03 PCR amplification products. (**B**): IFA identification performed with a monoclonal antibody against FAdV-4 Fiber-2 protein.

DNA extracted from liver tissue homogenates of diseased chickens served as the template for PCR using FAdV-4-specific primers, which yielded a specific 421 bp target band. The PCR-positive sample was inoculated onto CEL cells for viral isolation (Figure 1A). Following three serial blind passages, three rounds of plaque purification were carried out to generate a homogeneous virus isolate, designated strain GXYL-03. IFA verified the identity of this isolate: CEL cells infected with strain GXYL-03 produced positive signals when stained with an anti-FAdV-4 Fiber-2 monoclonal antibody, whereas uninfected mock control cells displayed no fluorescence (Figure 1B).

### 3.2. Pathogenicity Determination of Strain GXYL-03

**Figure 2:**
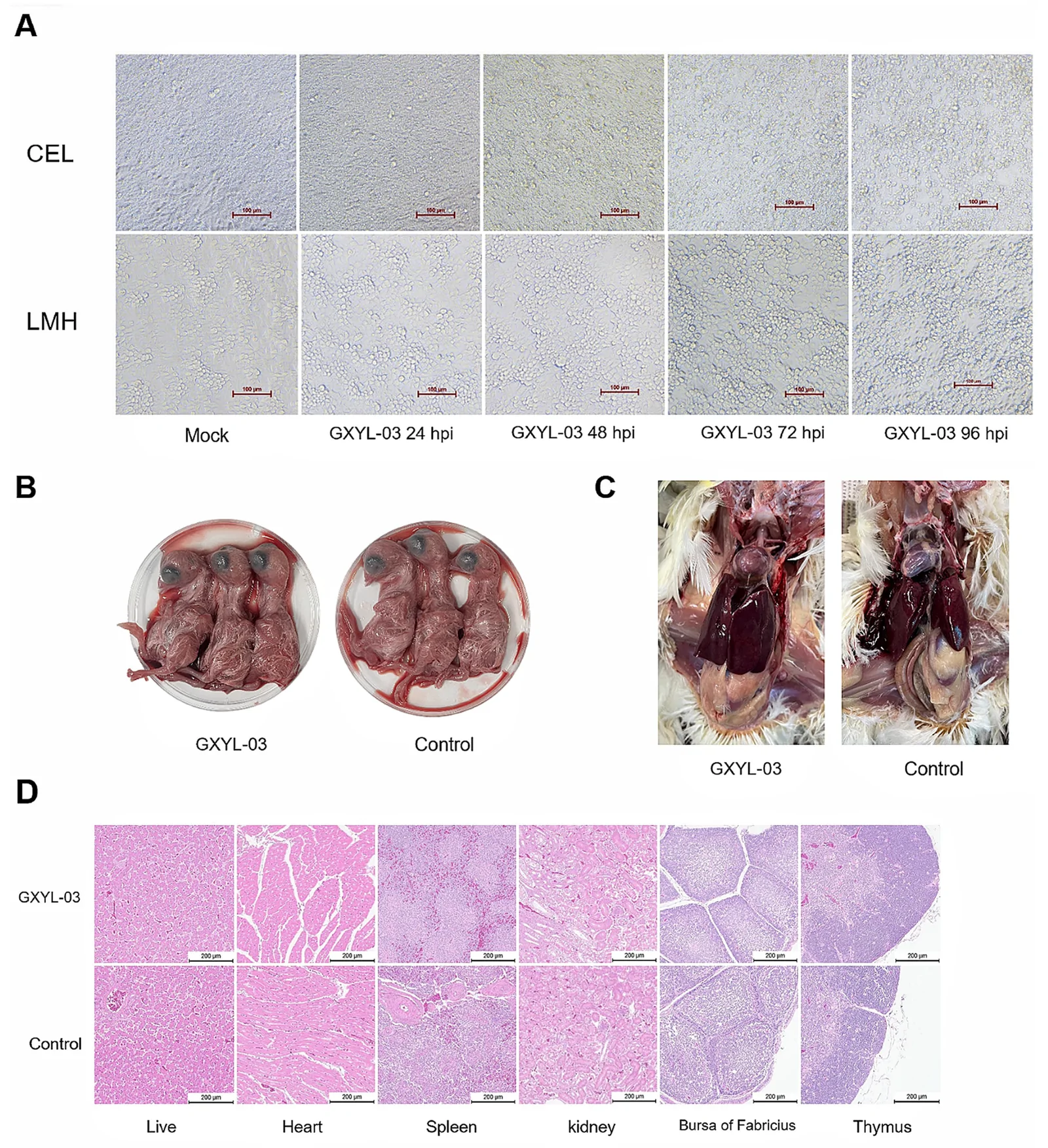
Pathogenicity assessment of strain GXYL-03. (**A**): Cytopathic effects induced by GXYL-03 in LMH and CEL cells. (**B**): Infection with GXYL-03 at 10^5^ TCID_50_ does not cause morbidity or mortality in chicken embryos. (**C**): Infection with GXYL-03 at 10^6^ TCID_50_ per bird fails to induce hydropericardium and hepatic lesions in SPF chickens. (**D**): Infection with GXYL-03 at 10^6^ TCID_50_ per bird does not result in the production of histological lesions in the liver, heart, spleen, kidney, bursa of Fabricius, or thymus.

To characterize the in vitro infectivity of strain GXYL-03, CEL and LMH cells were separately inoculated with the virus, and CPE was detected in both cell lines (Figure 2A). 10-day-old SPF chicken embryos were inoculated with strain GXYL-03 onto the CAM. No embryo deaths were recorded within 96 postinoculation, and compared with control embryos, infected embryos displayed no apparent swelling, atrophy, edema or hemorrhage (Figure 2B).

To assess the in vivo pathogenicity of this isolate in SPF chickens, 4-week-old birds were intramuscularly inoculated with serial dilutions of strain GXYL-03. This isolate was apathogenic to 4-week-old SPF chickens at a dose of 10^6^ TCID_50_, whereas obvious clinical manifestations and partial mortality occurred when the inoculation dose was elevated to 10^7^–10^8^ TCID_50_. No morbidity or mortality was recorded across groups dosed at 10^2^ to 10^6^ TCID_50_ throughout the observation period. In contrast, chickens given 10^7^ or 10^8^ TCID_50_ developed typical clinical signs starting at 4 dpi, including anorexia, ruffled feathers, lethargy, pale combs and wattles, and green watery diarrhea. In the 10^8^ TCID_50_ group, six of the ten birds died at 5–6 dpi. Clinical manifestations in surviving chickens gradually abated by 9 dpi, and all survivors recovered to a healthy state identical to that of the control group by 14 dpi (Table 2).

Chickens intramuscularly inoculated with strain GXYL-03 at 10^6^ TCID_50_ per bird were euthanized for necropsy. Gross pathological inspection failed to detect hallmark HHS lesions typical of highly pathogenic FAdV-4, such as amber pericardial effusion, hepatomegaly, jaundice and petechial hemorrhages; no distinguishable macroscopic changes were observed relative to those of the blank control birds (Figure 2C).

Histopathological analysis revealed unaltered myocardial tissue, with no obvious hyperplasia, vascular stenosis or inflammatory cell infiltration. The hepatic lobules maintained distinct boundaries, and no abnormal proliferation or inflammatory infiltration was visible within the portal tracts. The spleen exhibited regular cellular structures with clearly separated red and white pulp. The renal parenchyma remained intact, and the tubular epithelial cells showed no marked degeneration or necrosis. Lymphocytes in the bursa of Fabricius were orderly arranged, and lacked inflammatory infiltration, degeneration or necrotic lesions. The morphology of the proventricular mucosa remained intact, with no hemorrhagic or necrotic foci in the epithelial layers. Overall, no significant histopathological disparities in these organs were detected between infected and control animals (Figure 2D).

### 3.3. Evaluation of the Immune Protective Efficacy of Strain GXYL-03

**Figure 3:**
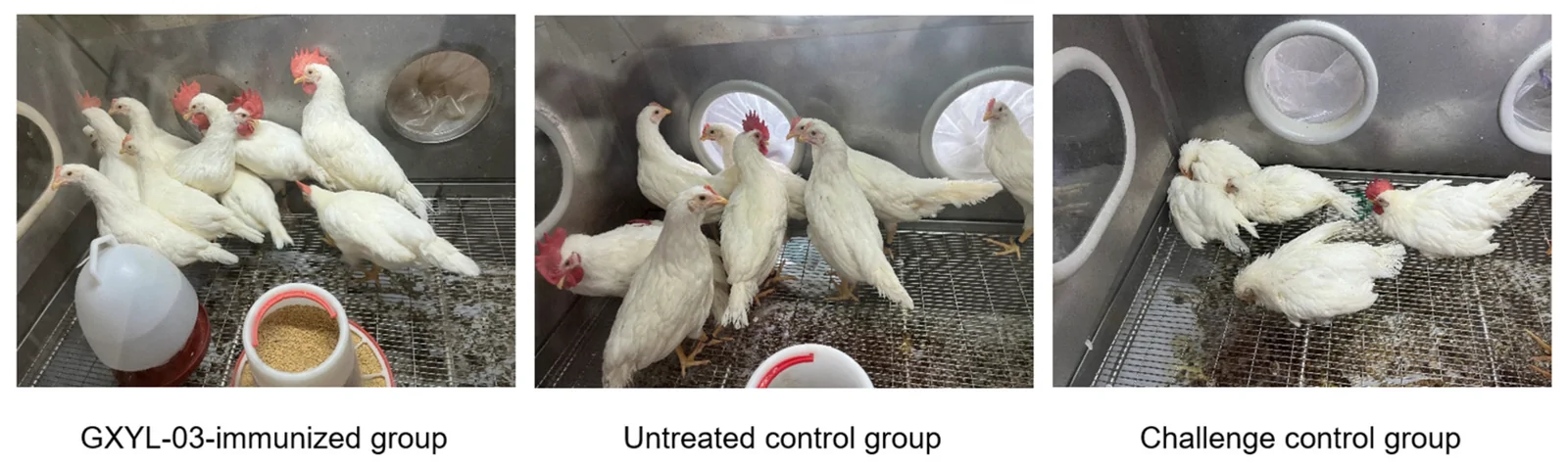
Immunoprotective efficacy of strain GXYL-03.

The birds in the immunization–challenge group were intramuscularly vaccinated with strain GXYL-03 at 5 × 10^4^ TCID_50_ per bird, followed by intramuscular challenge with the virulent strain GX2017-005 at 5 × 10^3^ TCID_50_ per bird 14 days postvaccination. Chickens in the challenge control group developed clinical signs starting at 3 dpc, and deaths first occurred at 4 dpc, with 100% mortality (10/10) reached by 7 dpc. In stark contrast, the immunized group exhibited no morbidity or mortality during the entire 14-day observation period, with activity and appetite levels identical to those of the blank control group (Figure 3).

### 3.4. Complete Genome Sequencing and Analysis of Strain GXYL-03

The complete genome of strain GXYL-03 is 45,781 bp in length (GenBank accession No. PV667637). Nucleotide composition analysis yielded 23.3% A, 21.99% T, 26.97% G and 27.74% C. A total of 44 ORFs were predicted across the full sequence.

**Figure 4:**
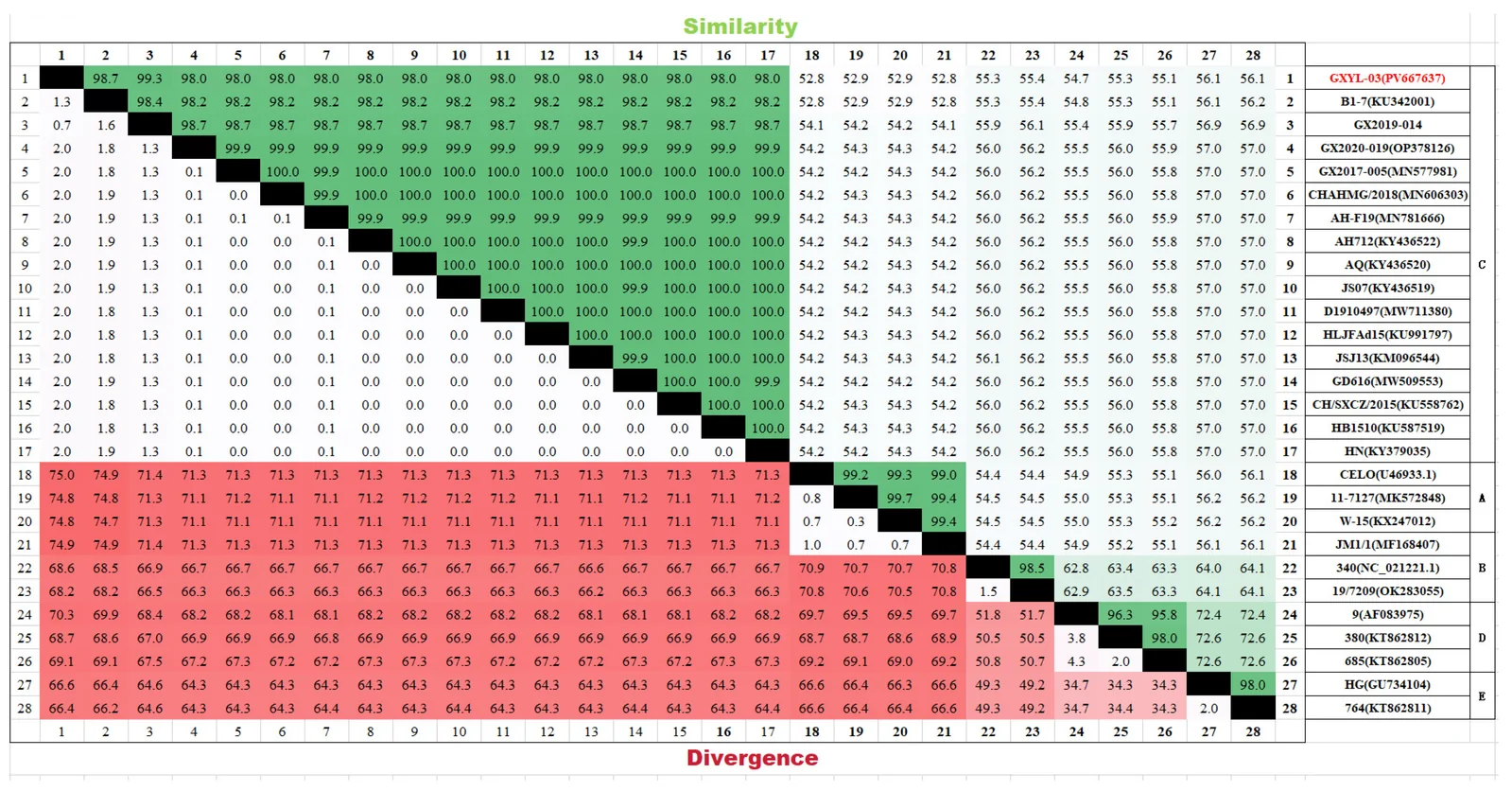
Whole-genome sequence identity analysis of strain GXYL-03. Strain GXYL-03 is highlighted in red. Darker colors indicate higher homology. Panels A–E correspond to Species A–E, respectively.

**Figure 5:**
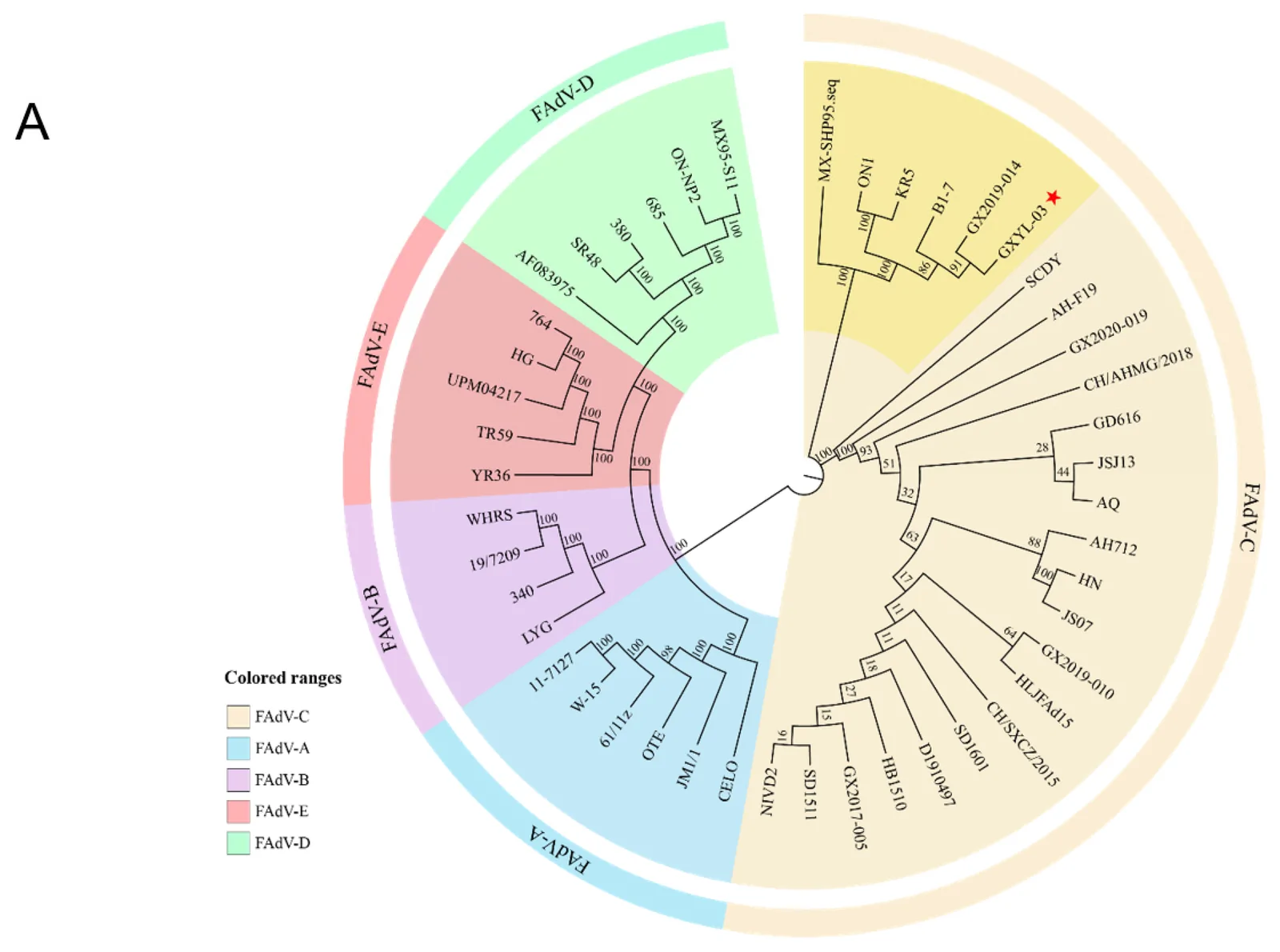

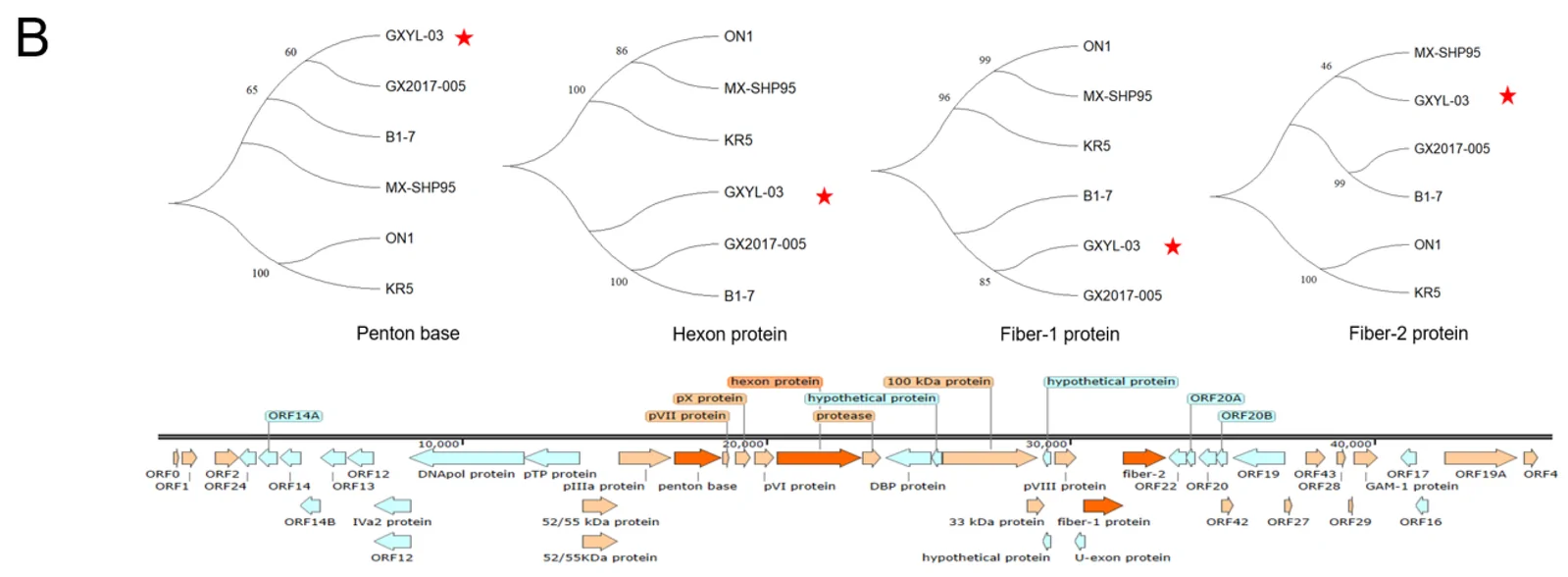
Phylogenetic analysis of FAdVs. (**A**): Phylogenetic tree constructed based on the complete genome nucleotide sequences of strain GXYL-03 and FAdV subgroup A–E reference strains. (**B**): Phylogenetic tree constructed based on the major structural proteins of strain GXYL-03 and FAdV-4 strains of different virulence levels. Strain GXYL-03 is indicated by a red asterisk. Colored arrows represent the locations of different ORFs in the full length genome.

Whole-genome sequence identity analysis showed that GXYL-03 shares 52.8%, 52.8–55.4%, 54.7–55.3%, and 56.1% identity with FAdV-A, FAdV-B, FAdV-D, and FAdV-E, respectively, indicating relatively distant genetic relationships with these species. Among FAdV-4 strains, GXYL-03 exhibits the highest identity with low-pathogenicity strains (99.3%), followed by non-pathogenic strains (98.7%), while it displays the lowest identity (98.0%) with highly pathogenic strains circulating in China since 2015 (Figure 4). Phylogenetic analysis further demonstrated that strain GXYL-03 belongs to serotype 4 of FAdV-C. In the whole-genome phylogenetic tree (Figure 5A), GXYL-03 clustered with the Chinese isolate GX2019-014, the Canadian strain ON1, the Mexican strain MX-SHP95, and the Korean strain KR5, whereas it was distantly related to the highly pathogenic strain GX2017-005 that has emerged in China in recent years. Notably, comparison of the major structural proteins revealed that GXYL-03 was more closely related to the highly pathogenic strain GX2017-005 in the Penton base, Hexon, and Fiber-1 proteins (Figure 5B).

### 3.5. Characterization of Polysome Profiling in Chicken Livers Infected with FAdV-4 Strains of Different Virulence

**Figure 6:**
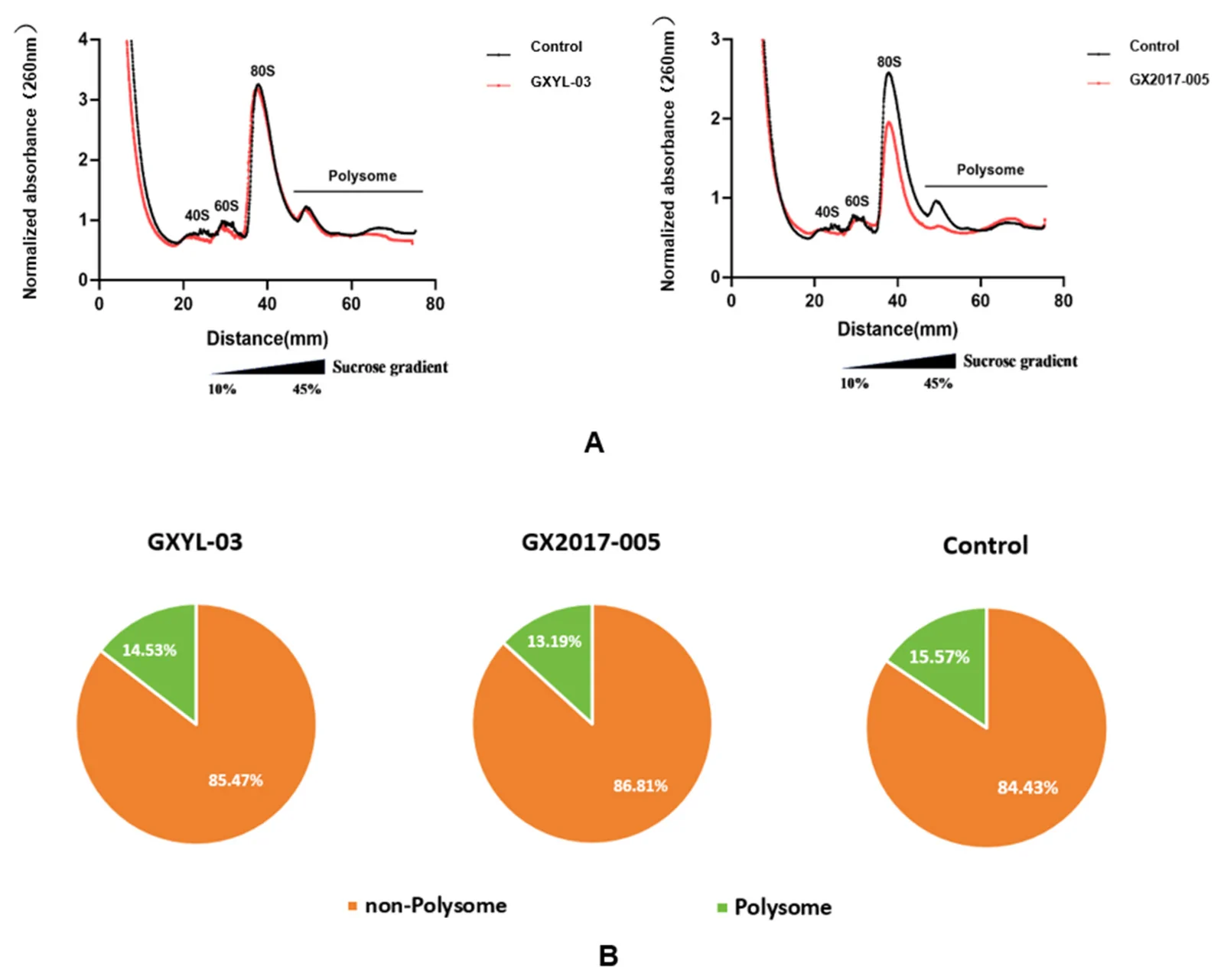
Polysome profiling analysis of liver tissues from FAdV-4-infected SPF chickens. (**A**): Normalized ribosome distribution curves based on absorbance at the 260 nm. (**B**): Pie charts showing the statistics of P/N ratio in samples from each group. The P/N ratio was calculated according to the AUC of the polysome profiles to evaluate the global translational activity of cells.

All the samples displayed well-resolved characteristic ribosomal peaks, indicating efficient ribosome fractionation (Figure 6A). Following area under the curve (AUC) normalization of each sample, the polysome-to-nonpolysome (P/NP) ratio was computed to evaluate global translational activity. No significant differences were detected among the GXYL-03, GX2017-005, and control groups (*p* > 0.05), suggesting that infection did not alter the global translation flux (Figure 6B). Notably, compared with the control group, the GX2017-005 group exhibited pronounced differences in peak shape and area in the 80S monosome and disome regions, implying that GX2017-005 infection induced a redistribution of ribosome assembly patterns within cells.

### 3.6. Overview of High-Throughput Sequencing of Chicken Livers Infected with FAdV-4 Strains of Different Virulence

**Figure 7:**
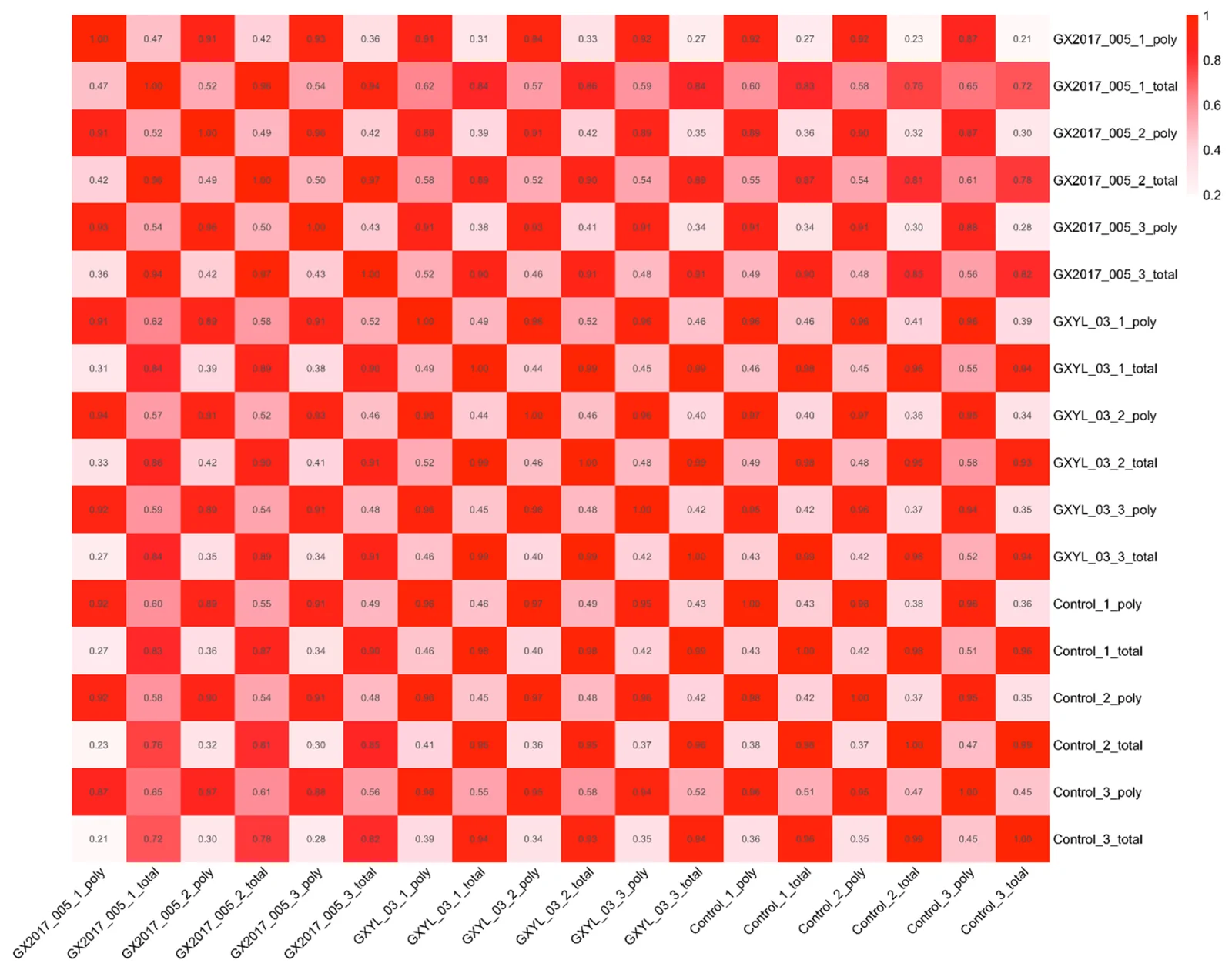
Pearson correlation analysis of RNA-seq and Polysome-seq data across samples.

RNA-seq and polysome-seq libraries were constructed for liver samples from the GXYL-03 infection group, GX2017-005 infection group, and control group, and the high-quality clean reads were aligned to the chicken reference genome *Gallus gallus* GRCg7b. For polysome-seq, the clean read counts ranged from 34.79 to 42.12 million, 34.58 to 35.85 million, and 29.77 to 43.29 million for the GXYL-03, GX2017-005, and control groups, respectively, with corresponding genome mapping rates of 88.59–91.51%, 74.30–83.88%, and 85.42–91.80%, respectively. In the matching RNA-seq libraries, the numbers of clean reads were 35.76–48.03 million, 32.90–37.40 million, and 32.13–37.92 million across the three groups, and the alignment rates reached 87.47–89.28%, 86.62–91.26%, and 89.41–92.23%, respectively. Pearson correlation analysis demonstrated strong concordance among biological replicates within both the control and FAdV-4 infection groups (Figure 7), confirming reliable sample reproducibility and warranting subsequent integrated analysis at the transcriptional and translational levels.

### 3.7. Transcriptomic and Translatomic Differential Expression and Integrated Analysis of Transcription–TE in FAdV-4-Infected SPF Chicken Livers

To elucidate the regulatory features of host gene expression at the transcriptional and translational levels during FAdV-4 infection, DEGs were screened based on transcriptome, translatome, and TE profiling in this study. A total of 2131 transcriptomic DEGs were identified in the GXYL-03-infected groups compared with the control group, whereas only 71 translatomic DEGs were detected. For the GX2017-005 infection group, 7767 transcriptomic DEGs and 148 translatomic DEGs were obtained. Both FAdV-4 strains exhibited substantially more DEGs at the transcriptional level than at the translational level, indicating that viral infection induces widespread transcriptional perturbations, accompanied by a significant translational buffering effect. Further TE analysis identified 278 TE-altered genes in the GXYL-03 group, including 115 upregulated and 163 downregulated genes, and 346 TE-altered genes in the GX2017-005 group, consisting of 132 upregulated and 214 downregulated genes. Collectively, these results demonstrate that FAdV-4 infection specifically modulates the TE of a subset of host genes, predominantly suppressing translation. These findings suggest that FAdV-4 infection inhibits host gene expression mainly at the translational level.

**Figure 8:**
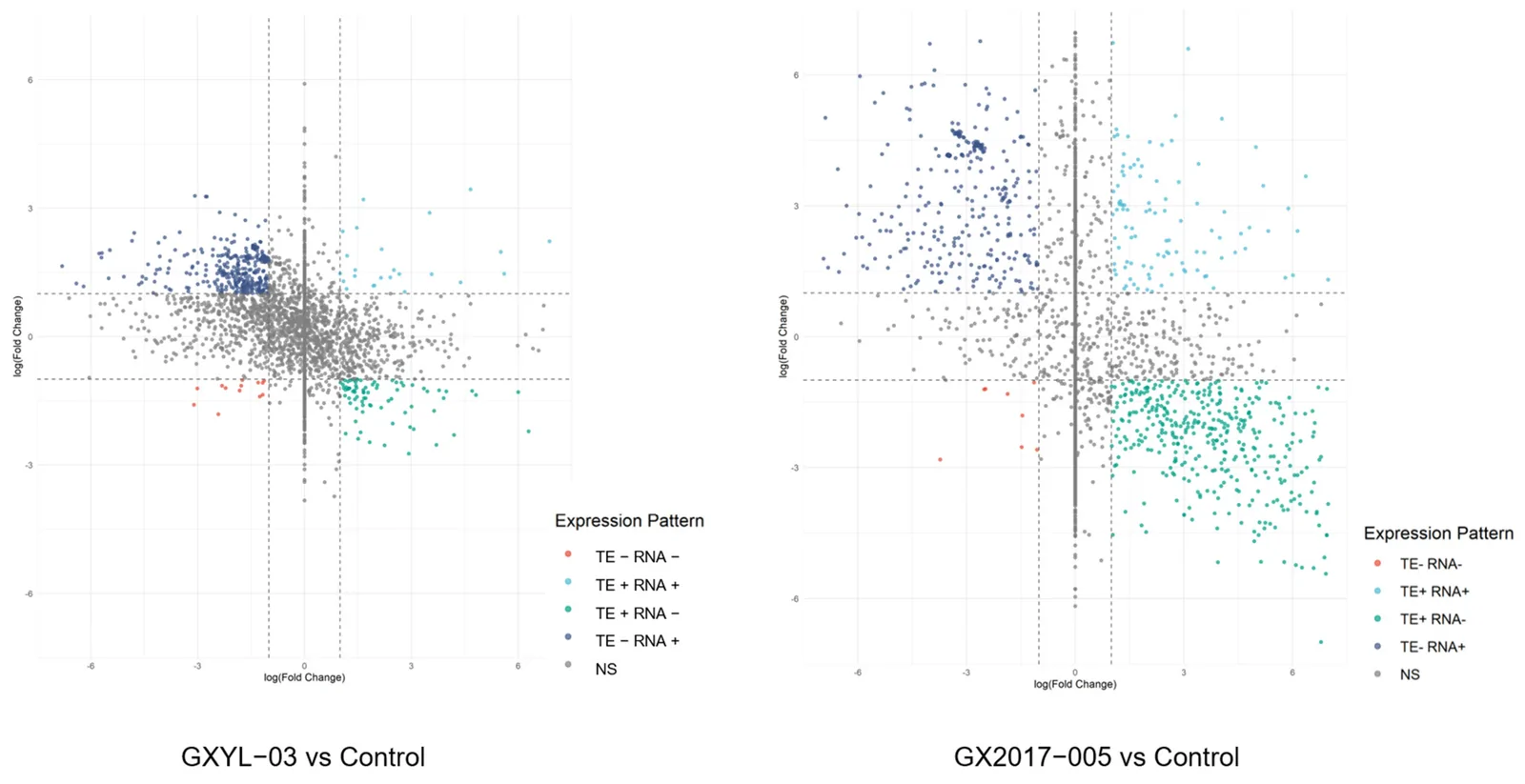
Combined scatter plot of changes in host transcript levels and TE upon FAdV-4 infection.

A combined scatter plot was constructed to illustrate host alterations in transcription levels and TEs upon FAdV-4 infection. The y-axis shows the transcriptomic log2 (FC) of genes, and the x-axis shows the log2 (FC) of the TE. Dotted lines denote the differential cutoff threshold of |log2FC| = 1. With respect to the GXYL-03 strain with reduced virulence, 89.6% of genes exhibited no significant changes. The proportions of genes in the TE+RNA+ and TE+RNA− quadrants were both 1.7%, with TE−RNA+ accounted for 7.8% and TE−RNA− accounted for 0.2%. In the high-virulence GX2017-005 group, 77.1% of the genes showed no significant changes in the expression, with 2.7% for TE+RNA+, 11.3% for TE+RNA−, 8.5% for TE−RNA+, and 0.2% for TE−RNA− (Figure 8).

**Figure 9:**
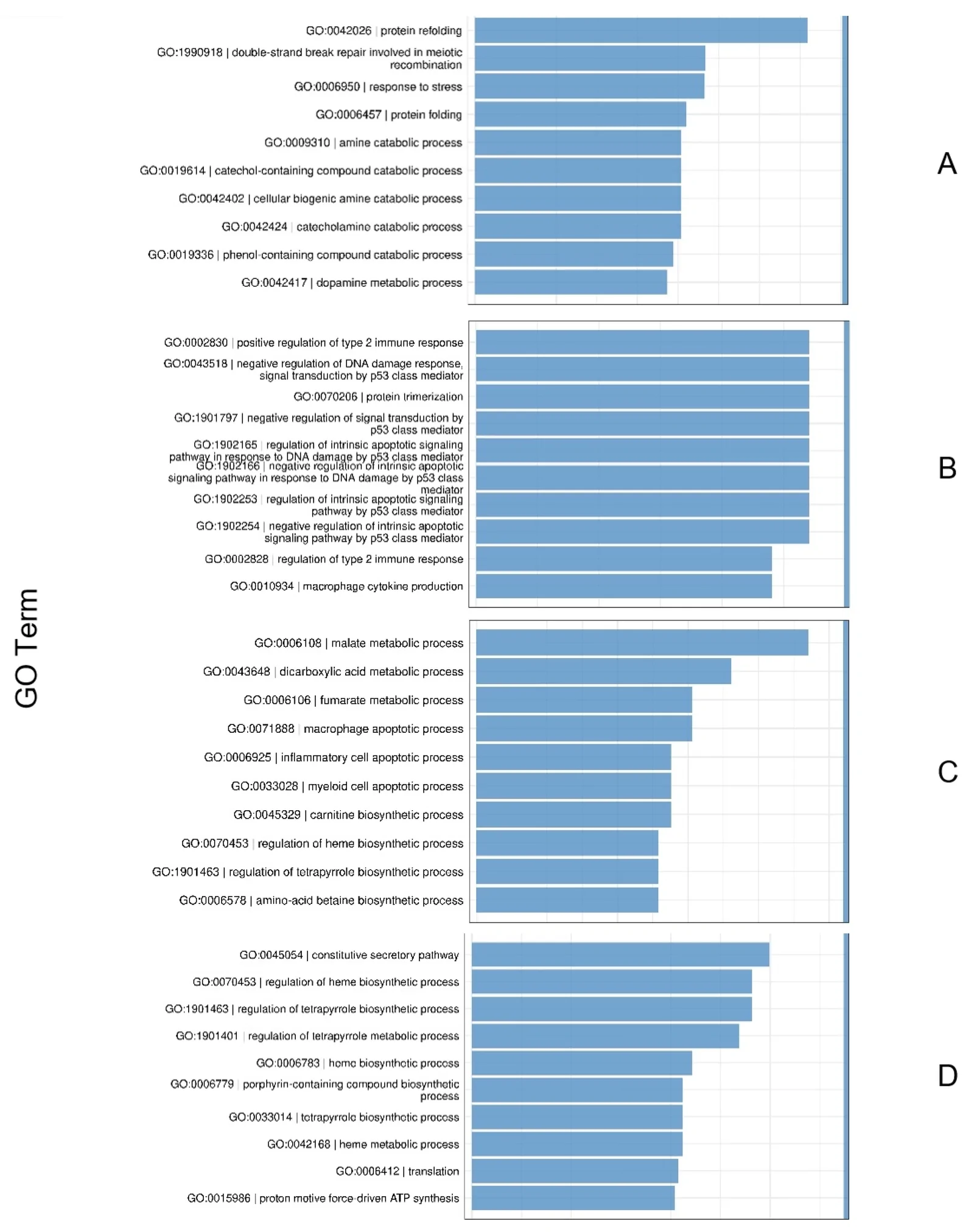
GO-biological process enrichment analysis of TE+RNA+ and TE−RNA− quadrant-specific DESs in chicken livers infected with FAdV-4 strains of different virulence. (**A**): GO enrichment of TE+RNA+ DESs for the GXYL-03 strain. (**B**): GO enrichment of TE+RNA+ DESs for the GX2017-005 strain. (**C**): GO enrichment of TE−RNA− DESs for the GXYL-03 strain. (**D**): GO enrichment of TE−RNA− DESs for the GX2017-005 strain.

To explore the coordinated functions of transcription and translation, we performed GO biological-process enrichment analysis on the DEGs across the four quadrants. With respect to the GXYL-03 strain with reduced virulence, TE+RNA+ DEGs were predominantly protein-coding genes enriched in cellular stress response, DNA-damage repair, vesicular transport, and protein homeostasis, including *HSPA2*, *HSPB9*, *FANCD2*, *EHD2*, and *Vault* (Figure 9A, Table S1). In the highly pathogenic GX2017-005 strain, TE+RNA+ DEGs included protein-coding genes associated with immune–inflammatory responses, autophagy, and mitochondrial stress (*CD74*, *ATG2B*, *FKBP5*, *ND1*, *COX1*), as well as abundant noncoding RNAs such as snoRNAs, snRNAs, and chicken-derived miRNAs. This observation suggests that infection by the high-virulence strain simultaneously activates host ribosome-modification and mRNA-splicing regulatory systems to drive widespread RNA-level reprogramming (Figure 9B, Table S2). Notably, *SNORD15*, *Vault*, and *HSPB9* represented shared TE+RNA+ DEGs in both strains. The Vault ribonucleoprotein complex mediates host innate immunity and modulates the type-I interferon pathway. As a small heat-shock protein, HSPB9 mitigates virus-triggered hepatocyte damage by preserving protein homeostasis and suppressing apoptosis, making it a core conserved host responder to FAdV-4 infection.

TE−RNA− DEGs from GXYL-03 included the complement-regulatory gene *CFI*, hypoxia-responsive transcription factor *EPAS1*, and critical genes governing hepatic glucose-lipid and energy metabolism (*ME1*, *FH*, *BBOX1*). These data demonstrate that infection with the strain with reduced virulence substantially disrupts hepatic complement immune homeostasis and basal metabolism (Figure 9C, Table S3). The highly pathogenic GX2017-005 yielded very few TE−RNA− DEGs, which encoded mainly ribosomal structural proteins (*RPS12* and *RPL23*) and mitochondrial function-associated factors (*ATP5C1* and *CHCHD10*) (Figure 9D, Table S4).

**Figure 10:**
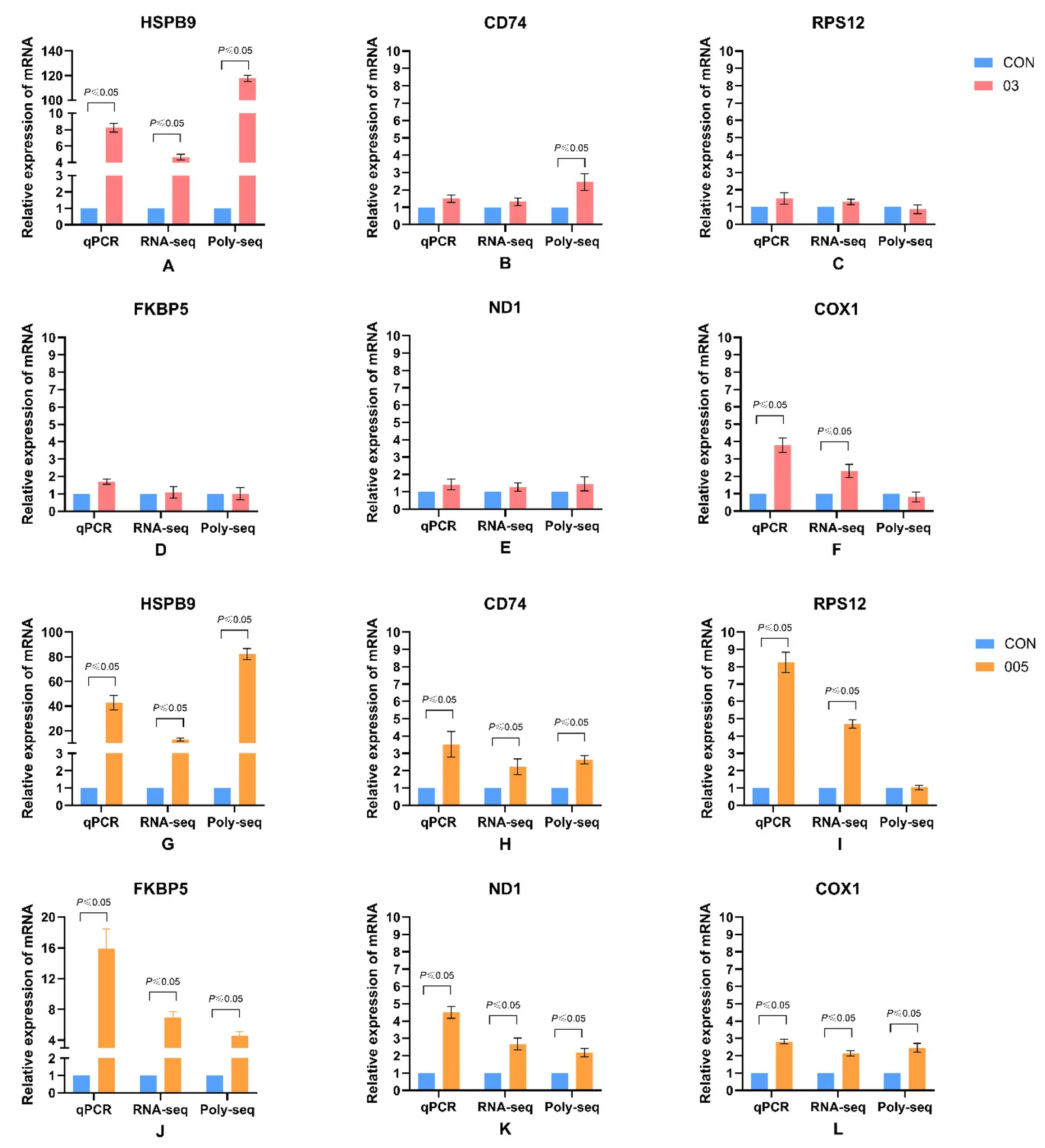
Consistency validation of six representative genes by qPCR versus RNA-seq/Polysome-seq. (**A**–**F**): Comparison of the relative expression levels of six genes, namely, HSPB9 (**A**), CD74 (**B**), RPS12 (**C**), FKBP5 (**D**), ND1 (**E**), and COX1 (**F**), in the GXYL-03 infection group; (**G**–**L**): Comparison of the relative expression levels of six genes, namely, HSPB9 (**G**), CD74 (**H**), RPS12 (**I**), FKBP5 (**J**), ND1 (**K**), and COX1 (**L**), in the GX2017-005 infection group. GAPDH and β-actin were used as dual internal reference genes, and the relative qPCR expression levels were calculated using the 2^−ΔΔCt^ method. The connected lines in the figure indicate *p* ≤ 0.05, representing statistically significant differences compared with the control group.

Based on the quadrant classification and functional enrichment results, six representative DEGs, including the cellular stress gene *HSPB9*, the immune–inflammatory genes *CD74* and *FKBP5*, the ribosomal 40S gene *RPS12*, and mitochondrial-encoded genes *ND1* and *COX1*, were selected for qPCR validation. The expression trends of these genes in the GXYL-03 and GX2017-005 infected groups were highly consistent with the RNA-seq results (Figure 10), confirming the reliability of our transcriptomic sequencing data.

## 4. Discussion

FAdV-4 is the major pathogen responsible for HHS in poultry [32]. Highly pathogenic FAdV-4 strains circulating in China exhibit strong virulence, high mortality and wide transmission [18,33,34], resulting in substantial economic losses to the poultry industry. Elucidating the pathogenic mechanisms of FAdV-4 strains with distinct virulence and exploring the biological advantages of low-virulence strains are critical for understanding viral pathogenesis and developing novel vaccines. Previous studies have constructed a virulence gene deleted FAdV-4 strain rHN20 and developed an FAdV-4-vvIBDV bivalent vector vaccine based on this backbone [35,36].

In this study, strain GXYL-03 was obtained by PCR identification, cell isolation–culture and plaque purification. IFA confirmed the expression of the FAdV-4 signature Fiber-2 protein. In vitro assays revealed that GXYL-03 efficiently infected CEL and LMH cells and induced typical CPE, indicating competent in-vitro replication capacity. Nevertheless, in-vivo animal experiments revealed its markedly attenuated virulence. High-dose inoculation caused no mortality or obvious histological lesions in the chicken embryos. In gradient-dose infection assays using SPF chickens, the routine immunization dose of 10^6^ TCID_50_ triggered no clinical signs. Only ultra-high doses (10^7^–10^8^ TCID_50_) induced mild transient symptoms, and all animals recovered without persistent tissue injury. Necropsy and histopathological examinations revealed no HHS-characteristic lesions including hepatic hemorrhage, edema and hydropericardium. The heart, spleen, kidney, bursa of Fabricius and other organs retained intact structures without obvious inflammatory infiltration or cellular necrosis. These results demonstrated that GXYL-03 is a naturally occurring FAdV-4 strain with reduced virulence, whose attenuated phenotype was further explained by whole-genome sequencing and phylogenetic analysis. Importantly, immunoprotection assays verified that GXYL-03 conferred complete protection against lethal challenge with the highly prevalent pathogenic strain GX2017-005; no disease or death occurred in immunized chickens, suggesting favorable immunogenicity and great potential as a live-attenuated vaccine candidate.

Polysome profiling revealed that infection with both high- and reduced-virulence FAdV-4 did not alter the global translational flux of host cells, with no significant change in the P/NP ratio, suggesting that FAdV-4 does not suppress host global translation. However, infection with the high-virulence strain GX2017-005 remodeled the assembly patterns of 80S monoribosomes and disomes. These results indicate that the highly virulent virus specifically remodels ribosome arrangement to target the translation of a subset of host genes rather than causing global translational inhibition, representing a novel regulatory feature of highly pathogenic FAdV-4.

Integrated transcriptome–translatome analysis identified a prominent transcription–translation buffering effect during FAdV-4 infection, which constitutes the core innovation of this study. The transcription–translation buffering effect has been widely reported in eukaryotes including yeast, mammals and plants [37,38,39]. When substantial fluctuations occur at the transcriptional level, TE produces compensatory reverse changes to buffer alterations in mRNA abundance, thereby stabilizing ribosome-binding levels and protein output to maintain homeostatic gene expression. In the present study, infections by both strains triggered large-scale transcriptional perturbations in host cells. The high-virulence strain resulted in the expression of 7767 DEGs at the transcriptional level, whereas only hundreds of genes were differentially expressed at the translational level. A large proportion of transcriptional disturbances were buffered and offset at the translational stage. As a host self-protective strategy, this mechanism alleviates the proteome imbalance caused by transcriptional disorders and maintains the physiological homeostasis of hepatocytes.

Four-quadrant stratified enrichment analysis of TE revealed distinct host response patterns induced by the two strains. Upon infection with GXYL-03, a strain with reduced virulence, the host preferentially activated protein homeostasis, DNA-damage repair and cellular stress pathways centered on *HSPB9*, *Vault* and *HSPA2*. Moderate host defense was achieved by repairing damaged proteins, inhibiting apoptosis and eliminating cellular stress insults. Moreover, hepatic glycolipid and energy metabolism pathways were downregulated, which enhanced host tolerance by reducing cellular metabolic activity. Excessive inflammatory bursts were absent throughout the infection, resulting in no obvious pathological lesions. In contrast, infection with the highly pathogenic strain GX2017-005 markedly activated immune–inflammatory, autophagy and mitochondrial stress pathways, triggering severe hepatic inflammatory injury and mitochondrial dysfunction. Moreover, noncoding RNAs including snoRNAs, snRNAs and miRNAs, were highly enriched, and strongly regulated ribosome modification and mRNA splicing, initiated global RNA reprogramming, and amplified inflammatory disturbance and cellular damage, resulting in severe hepatic lesions and host death. Furthermore, the expression of HSPB9 and Vault, the core responsive genes shared by both strains, modulated the type-I interferon pathway and maintained hepatocyte homeostasis, serving as conserved key targets for host defense against FAdV-4 infection. The divergent responses of these pathways and core genes further explain the molecular basis underlying the distinct virulence phenotypes of the two FAdV-4 strains at the translational regulatory level, and highlight the decisive role of host cellular stress responses in determining the outcome of viral infection.

It should be acknowledged that the above pathway interpretations are based on gene set and four-quadrant TE enrichment analyses of hepatic transcriptomic and translatomic data, and do not prove that these pathways mediate the distinct virulence phenotypes of the two strains, nor that HSPB9 and Vault are functionally required for host defense. Meanwhile, the present conclusions are derived mainly from transcriptomic and translatomic data of the liver—the primary target organ of FAdV-4—and may not fully reflect the molecular responses occurring in the heart, kidneys or immune organs. Future multi-organ omics studies are required to systematically characterize the systemic host responses induced by FAdV-4 infection.

## 5. Conclusions

In this study, we identified a naturally attenuated FAdV-4 strain, GXYL-03, with genetic stability, high safety and favorable immune-protective efficacy, highlighting its great potential for live-attenuated vaccine development. Using genomic analysis, ribosome profiling and integrated transcriptome–translatome sequencing, we elucidated the differential pathogenic mechanisms of high- and reduced-virulence FAdV-4 and revealed the link between the transcription–translation buffering effect and viral virulence differentiation. The strain with reduced virulence maintains host homeostasis by activating mild cellular stress pathways, whereas the high-virulence strain induces host injury through RNA reprogramming, disrupted ribosome assembly and inflammatory imbalance. This work advances our understanding of FAdV-4 pathogenic mechanisms and provides an important theoretical basis and experimental support for fowl adenovirus prevention–control and novel live-attenuated vaccine development.

## Figures and Tables

**Table 1:** Sequences of the qPCR primers used in this study.

Genes	Forward Primer (5′-3′)	Reverse Primer (5′-3′)	Accession Number
HSPB9	ACGGAACAAACGGTCTGCATC	CATGATGGATGGAGCGTGTCT	NM_001010842.3
CD74	AGGCCAGTTTCGGTGGTGTCCA	AGTGCCCTCAATCCTGGTGCCA	NM_001001613.2
RPS12	GGGTCGTGGTGGGGTTATTC	TTCCCAAGTTGCAAACCGT	XM_046938860.1
FKBP5	TTGCTGAGAGGGATGCGAAG	ATACGTGCCCTCTGTCTCT	XM_015298716.4
ND1	CACCCAAGAGCCCATCTACCTCATT	TGGTCCGGCGGCATATTCTACATT	AP003318.1
COX1	TCACAGGCTTTACCCTACACCCATC	AGTGTGTAGGCGTCTGGGTAATCTG	AP003318.1
GAPDH *	GAGGGTAGTGAAGGCTGCTG	CATCAAAGGTGGAGGAATGG	NM_205518.1
β-actin	GCCCCCAAAGTTCTACAAT	AGGCGAGTAACTTCCTGTA	NM_204305.1

* The GAPDH primers were obtained from Dalgaard et al. (2015) [31].

**Table 2:** Pathogenicity of strain GXYL-03 in 4-week-old SPF chickens.

Group	Dose (mL)	Virus Titer TCID_50_/0.25 mL	Intramuscular Injection	
			Onset/Total	Death/Total
1	0.25	10^2^	0/10	0/10
2	0.25	10^3^	0/10	0/10
3	0.25	10^4^	0/10	0/10
4	0.25	10^5^	0/10	0/10
5	0.25	10^6^	0/10	0/10
6	0.25	10^7^	3/10	0/10
7	0.25	10^8^	6/10	6/10
Mock	0.25	PBS	0/10	0/10

## Data Availability

The original contributions presented in this study are included in the article/Supplementary Material. Further inquiries can be directed to the corresponding authors.

## Supplementary Materials

The following supporting information can be downloaded at: https://www.mdpi.com/article/10.3390/microorganisms14102226/s1. The data related to four-quadrant stratified enrichment analysis of TE in this study are provided in Supplementary Information S1: Table S1. GXYL-03 vs Control TE+RNA+ Differentially Expressed Genes; Table S2. GX2017-005 vs Control TE+RNA+ Differentially Expressed Genes; Table S3. GXYL-03 vs Control TE-RNA- Differentially Expressed Genes; Table S4. GX2017-005 vs Control TE-RNA- Differentially Expressed Genes. The raw sequence data reported in this paper have been deposited in the Genome Sequence Archive in National Genomics Data Center, China National Center for Bioinformation/Beijing Institute of Genomics, Chinese Academy of Sciences (GSA: CRA048760), and are publicly accessible at https://ngdc.cncb.ac.cn/gsa (accessed on 10 September 2026).
