# Supplementary material for: Integrated Transcriptome–Translatome Analysis Reveals Translational Buffering in the Liver of Chickens Infected with Fowl Adenovirus Serotype 4 Strain GXYL-03, a Strain with Reduced Virulence
Source: Microorganisms. 2026 Oct 2;14(10):2226. doi: 10.3390/microorganisms14102226 (PMC13707455; doi:10.3390/microorganisms14102226)
Supplement: Supplementary file 1 [file microorganisms-14-02226-s001.zip › microorganisms-4571042-supplementary.pdf]

Table S1　　GXYL-03 vs Control TE+RNA+ Differentially Expressed Genes

ENSEMBL	Regulated. Type_RNA	Gene.name RNA	logFC_RNA	Regulated. Type_polysome	Gene.name polysome	logFC_polysome	part
ENSGALG00010000013	Up		3.43640	Up		4.66015	TE+RNA+
ENSGALG00010000014	Up		3.20049	Up		1.64951	TE+RNA+
ENSGALG00010000015	Up		2.88605	Up		3.51049	TE+RNA+
ENSGALG00010000016	Up		2.53960	-		1.47045	TE+RNA+
ENSGALG00010002461	Up	TOMT	1.28889	-	TOMT	1.09222	TE+RNA+
ENSGALG00010003324	-		1.18673	-		1.9645	TE+RNA+
ENSGALG00010003346	Up		1.97304	-		5.51285	TE+RNA+
ENSGALG00010003395	Up		1.37876	-		2.41010	TE+RNA+
ENSGALG00010003401	Up		1.46758	-		1.00039	TE+RNA+
ENSGALG00010003408	Up		2.03715	-		2.16714	TE+RNA+
ENSGALG00010003420	Up		1.45779	-		2.66272	TE+RNA+
ENSGALG00010003799	-	SNORD15	1.45364	-	SNORD15	3.57166	TE+RNA+
ENSGALG00010008888	Up	FBXO46	1.18443	-	FBXO46	1.93275	TE+RNA+
ENSGALG00010010813	Up	EHD2	1.41124	-	EHD2	1.41155	TE+RNA+
ENSGALG00010012935	-	Vault	2.45376	-	Vault	1.07117	TE+RNA+
ENSGALG00010017470	Up	HSPA2	1.55438	Up	HSPA2	1.42311	TE+RNA+
ENSGALG00010017946	Up	PTGDR	1.36983	-	PTGDR	2.1466	TE+RNA+
ENSGALG00010023206	Up	HSPB9	2.22422	Up	HSPB9	6.8776	TE+RNA+
ENSGALG00010023860	Up	TRIP4	1.461908	-	TRIP4	5.60884	TE+RNA+
ENSGALG00010026166	Up	WNT9A	1.551249	-	WNT9A	2.50612	TE+RNA+
ENSGALG00010027361	Up	FANCD2	1.112492	-	FANCD2	1.18191	TE+RNA+
ENSGALG00010028316	Up	TIMM44	1.042290	Up	TIMM44	2.81023	TE+RNA+
ENSGALG00010028669	Up	ZBTB34	1.26386	-	ZBTB34	4.37966	TE+RNA+
ENSGALG00010029785	-		1.88733	-		1.19404	TE+RNA+

Table S2 GX2017-005 vs Control TE+RNA+ Differentially Expressed Genes

ENSEMBL	Regulated. Type_RNA	Gene.name RNA	logFC_RNA	Regulated. Type_polysome	Gene.name polysome	logFC_polysome	part
ENSGALG00010000004	Up		2.79687	-		1.03823	TE+RNA+
ENSGALG00010000006	Up		3.90384	-		1.32159	TE+RNA+
ENSGALG00010000007	Up	ND1	1.4214	-	ND1	1.12353	TE+RNA+
ENSGALG00010000010	Up		4.4565	-		2.05629	TE+RNA+
ENSGALG00010000013	-		1.44933	-		9.26792	TE+RNA+
ENSGALG00010000014	-		1.34998	-		5.79906	TE+RNA+
ENSGALG00010000015	-		1.30728	Up		6.98042	TE+RNA+
ENSGALG00010000016	Up		1.3918	Up		3.619225	TE+RNA+
ENSGALG00010000017	Up	COX1	1.10195	Up	COX1	1.35279	TE+RNA+
ENSGALG00010000021	Up		2.91845	-		1.32209	TE+RNA+
ENSGALG00010000031	Up		2.11813	Up		7.29155	TE+RNA+
ENSGALG00010000319	-		4.39182	-		2.39551	TE+RNA+
ENSGALG00010000753	Up	TRIO	1.90659	-	TRIO	2.5791	TE+RNA+
ENSGALG00010001442	Up	LDLRAD4	1.42301	-	LDLRAD4	2.02715	TE+RNA+
ENSGALG00010001914	Up		3.95864	-		3.40037	TE+RNA+
ENSGALG00010003455	Up		4.99366	-		4.04229	TE+RNA+
ENSGALG00010003798	Up	SNORD15	4.49219	Up	SNORD15	2.65894	TE+RNA+
ENSGALG00010003799	Up	SNORD15	3.45845	-	SNORD15	1.17316	TE+RNA+
ENSGALG00010004674	-	SNORA31	1.93001	-	SNORA31	7.97424	TE+RNA+
ENSGALG00010005209	-	gga-mir-1650	2.90071	-	gga-mir-1650	2.05774	TE+RNA+
ENSGALG00010005938	Up		3.87549	-		1.52377	TE+RNA+
ENSGALG00010007115	Up		1.58511	-		1.37646	TE+RNA+
ENSGALG00010007174	-		3.14875	-		2.49741	TE+RNA+
ENSGALG00010007502	-		1.66223	-		1.08096	TE+RNA+
ENSGALG00010007788	Up	PALM2AKAP2	4.62363	-	PALM2AKAP2	1.16696	TE+RNA+
ENSGALG00010007813	Up	U2	3.02816	-	U2	1.22612	TE+RNA+
ENSGALG00010007816	Up	U2	3.00872	-	U2	1.310787	TE+RNA+
ENSGALG00010007820	Up	U2	3.06558	-	U2	1.21553	TE+RNA+
ENSGALG00010007822	Up	U2	2.90754	-	U2	1.15232	TE+RNA+
ENSGALG00010007823	Up	U2	2.905371	-	U2	1.44145	TE+RNA+
ENSGALG00010007824	Up	U2	3.01207	-	U2	1.3578	TE+RNA+
ENSGALG00010007825	Up	U2	3.10511	-	U2	1.24292	TE+RNA+
ENSGALG00010007827	Up	U2	3.0302	-	U2	1.27721	TE+RNA+
ENSGALG00010007975	Up	ANKRD11	1.42281	-	ANKRD11	2.66117	TE+RNA+
ENSGALG00010008321	Up	ZFHX3	2.19445	-	ZFHX3	2.51425	TE+RNA+
ENSGALG00010008401	Up	PSMD8	1.074071	-	PSMD8	1.9875	TE+RNA+
ENSGALG00010008743	-		2.49754	Up		4.80484	TE+RNA+
ENSGALG00010008915	Up	Y_RNA	2.42951	-	Y_RNA	3.76626	TE+RNA+

ENSGALG00010009123	Up		1.52205	-		1.08807	TE+RNA+
ENSGALG00010009419	-		2.41559	-		5.32966	TE+RNA+
ENSGALG00010010808	-		2.64909	-		2.913	TE+RNA+
ENSGALG00010010865	Up		1.87755	Up		2.28914	TE+RNA+
ENSGALG00010011396	Up	SNORA74	3.78242	-	SNORA74	1.29752	TE+RNA+
ENSGALG00010011429	Up	UBAP2	1.68687	-	UBAP2	2.95125	TE+RNA+
ENSGALG00010011848	Up	U1	6.59577	Up	U1	3.12056	TE+RNA+
ENSGALG00010012935	Up	Vault	4.18641	-	Vault	1.34291	TE+RNA+
ENSGALG00010013450	Up	CD74	1.15953	-	CD74	1.40708	TE+RNA+
ENSGALG00010014133	-	SNORA72	2.26296	-	SNORA72	3.98785	TE+RNA+
ENSGALG00010014223	Up	U12	5.06225	-	U12	2.77156	TE+RNA+
ENSGALG00010014431	Up		4.75052	-		1.13837	TE+RNA+
ENSGALG00010014871	-		2.20595	-		2.58638	TE+RNA+
ENSGALG00010015590	-	SNORA66	3.67422	-	SNORA66	1.79086	TE+RNA+
ENSGALG00010015614	-	ATG2B	1.02908	-	ATG2B	2.12139	TE+RNA+
ENSGALG00010015878	-	SNORD83A	2.2965	-	SNORD83A	1.65631	TE+RNA+
ENSGALG00010017380	Up	MARCKSL1	3.06146	-	MARCKSL1	2.03475	TE+RNA+
ENSGALG00010017644	Up	RBBP6	2.26964	-	RBBP6	1.54908	TE+RNA+
ENSGALG00010018021	Up	ATRN	2.88268	-	ATRN	2.47157	TE+RNA+
ENSGALG00010018475	-	SNORA28	1.94173	Up	SNORA28	3.35129	TE+RNA+
ENSGALG00010018542	Up	ZMIZ2	1.32973	-	ZMIZ2	2.04938	TE+RNA+
ENSGALG00010018773	Up		1.371678	-		3.57368	TE+RNA+
ENSGALG00010018864	Up	ABCA3	1.09669	-	ABCA3	1.72633	TE+RNA+
ENSGALG00010018964	-	SNORA63	2.36141	-	SNORA63	4.57452	TE+RNA+
ENSGALG00010018978	-		1.70974	-		1.43043	TE+RNA+
ENSGALG00010019156	-		3.4562	Up		5.18929	TE+RNA+
ENSGALG00010019886	-		4.05813	-		1.73572	TE+RNA+
ENSGALG00010019890	Up		4.5875	-		1.07781	TE+RNA+
ENSGALG00010020073	-	SNORA62	1.52592	Up	SNORA62	3.00581	TE+RNA+
ENSGALG00010020078	-	SNORA62	2.48158	-	SNORA62	1.57195	TE+RNA+
ENSGALG00010020480	Up	Metazoa_SRP	8.64982	-	Metazoa_SRP	1.45118	TE+RNA+
ENSGALG00010020797	-	DGCR8	1.51998	-	DGCR8	3.36953	TE+RNA+
ENSGALG00010020891	-	gga-mir-3540	1.93001	-	gga-mir-3540	4.41021	TE+RNA+
ENSGALG00010021045	Up	DPP4	1.57982	-	DPP4	2.10005	TE+RNA+
ENSGALG00010021095	Up	NT5C2	1.19439	-	NT5C2	1.62019	TE+RNA+
ENSGALG00010021711	Up		3.69552	-		1.3381	TE+RNA+
ENSGALG00010023044	-	gga-mir-3533	1.93001	Up	gga-mir-3533	3.56227	TE+RNA+
ENSGALG00010023206	Up	HSPB9	3.67413	-	HSPB9	6.3634	TE+RNA+
ENSGALG00010024094	-	SNORA23	2.86713	Up	SNORA23	1.95872	TE+RNA+
ENSGALG00010024481	-	SNORD21	1.62433	Up	SNORD21	1.88379	TE+RNA+
ENSGALG00010024532	-	TMEM86A	1.16953	Up	TMEM86A	2.38460	TE+RNA+

ENSGALG00010025276	Up	SNORA71	2.93261	-	SNORA71	5.8819	TE+RNA+
ENSGALG00010025444	Up		2.0362	-		1.19996	TE+RNA+
ENSGALG00010025572	Up		1.6822	-		1.5457	TE+RNA+
ENSGALG00010025756	-		1.93893	-		2.79455	TE+RNA+
ENSGALG00010025761	-	SNORA50A	2.85591	Up	SNORA50A	4.11453	TE+RNA+
ENSGALG00010025923	-	gga-mir-6619	2.41559	-	gga-mir-6619	6.13528	TE+RNA+
ENSGALG00010026369	Up	SNORD14	2.01877	Up	SNORD14	4.68928	TE+RNA+
ENSGALG00010026439	Up		3.93465	Up		1.62416	TE+RNA+
ENSGALG00010026647	-		3.05298	Up		3.36783	TE+RNA+
ENSGALG00010026649	-		1.3154	Up		1.51731	TE+RNA+
ENSGALG00010026668	Up		6.73172	-		1.04479	TE+RNA+
ENSGALG00010026893	Up	FKBP5	2.83062	-	FKBP5	2.20239	TE+RNA+
ENSGALG00010026900	Up		2.5063	-		1.39872	TE+RNA+
ENSGALG00010026902	-		1.93893	-		2.52817	TE+RNA+
ENSGALG00010026946	-		3.90315	Up		1.83369	TE+RNA+
ENSGALG00010027328	Up	RBM15	1.49031	-	RBM15	3.03248	TE+RNA+
ENSGALG00010027678	-		4.34513	-		4.98153	TE+RNA+
ENSGALG00010028398	-	SNORA84	4.58292	Up	SNORA84	1.452257	TE+RNA+
ENSGALG00010028923	Up		1.80101	-		4.0363	TE+RNA+
ENSGALG00010029120	-		3.32438	-		1.05777	TE+RNA+
ENSGALG00010029123	-		3.55984	-		2.24832	TE+RNA+
ENSGALG00010029127	-	SNORA65	1.40447	Up	SNORA65	6.00381	TE+RNA+
ENSGALG00010029384	-	WDR81	1.10816	-	WDR81	3.81469	TE+RNA+
ENSGALG00010029896	-		1.83919	-		2.44716	TE+RNA+
ENSGALG00010030085	-	U6	2.25347	-	U6	1.09324	TE+RNA+
ENSGALG00010030088	Up	SNORA50C	3.53867	-	SNORA50C	2.85901	TE+RNA+

Table S3 GXYL-03 vs Control TE-RNA- Differentially Expressed Genes

ENSEMBL	Regulated. Type_RNA	Gene. name RNA	logFC_RNA	Regulated. Type_polysome	Gene. name polysome	logFC_polysome	part
ENSGALG00010002972	Down	ME1	-1.08019	-	ME1	-1.30223	TE-RNA-
ENSGALG00010003845	Down	CFI	-1.1502	-	CFI	-1.77935	TE-RNA-
ENSGALG00010009103	Down	AP3S1	-1.21701	-	AP3S1	-3.00989	TE-RNA-
ENSGALG00010009197	Down	FH	-1.2033	-	FH	-2.21183	TE-RNA-
ENSGALG00010010850	Down		-1.36054	-		-1.17426	TE-RNA-
ENSGALG00010014347	-	KIAA0040	-1.8168	-	KIAA0040	-2.41956	TE-RNA-
ENSGALG00010017829	-	TRNP1	-1.59603	-	TRNP1	-3.10472	TE-RNA-
ENSGALG00010018414	Down	EPAS1	-1.40352	-	EPAS1	-1.25456	TE-RNA-
ENSGALG00010019123	Down	PLEKHO2	-1.26098	-	PLEKHO2	-1.81874	TE-RNA-
ENSGALG00010019820	Down	BBOX1	-1.02067	Down	BBOX1	-1.74558	TE-RNA-
ENSGALG00010021790	Down	PHF21A	-1.14988	-	PHF21A	-2.31616	TE-RNA-
ENSGALG00010022451	Down	CHP1	-1.02942	-	CHP1	-1.13324	TE-RNA-
ENSGALG00010026132	Down	DDTL	-1.08353	-	DDTL	-1.17123	TE-RNA-

Table S4 GX2017-005 vs Control TE-RNA- Differentially Expressed Genes

ENSEMBL	Regulated. Type_RNA	Gene.name RNA	logFC_RNA	Regulated. Type_polysome	Gene.name polysome	logFC_polysome	part
ENSGALG00010002667	Down		-2.53247	-		-1.47976	TE-RNA-
ENSGALG00010004255	Down	RPS12	-1.3179	Down	RPS12	-1.86698	TE-RNA-
ENSGALG00010005283	Down	ATP5C1	-1.8117	-	ATP5C1	-1.45641	TE-RNA-
ENSGALG00010005494	Down	PPDPFL	-2.59328	-	PPDPFL	-1.05391	TE-RNA-
ENSGALG00010010850	Down		-2.81704	Down		-3.72286	TE-RNA-
ENSGALG00010022173	Down	RPL23	-1.05201	-	RPL23	-1.138	TE-RNA-
ENSGALG00010025985	Down	CHCHD10	-1.19785	-	CHCHD10	-2.47719	TE-RNA-
ENSGALG00010027741	Down	RAB11B	-1.21227	-	RAB11B	-2.51032	TE-RNA-
